# Magnetic resonance-guided focused ultrasound surgery (MRgFUS) treatment for uterine fibroids

**DOI:** 10.2349/biij.6.2.e15

**Published:** 2010-04-01

**Authors:** BJJ Abdullah, RV Subramaniam, SS Omar, P Wragg, N Ramli, AL Wui, CC Lee, Y Yusof

**Affiliations:** 1 Department of Biomedical Imaging, University of Malaya, Kuala Lumpur, Malaysia; 2 Department of Obstetrics and Gynaecology, University of Malaya, Kuala Lumpur, Malaysia; 3 Insightec, United States

**Keywords:** MRgFUS, fibroids, technique

## Abstract

Magnetic Resonance-guided focused Ultrasound Surgery (MRgFUS) is gaining popularity as an alternative to medical and surgical interventions in the management of symptomatic uterine fibroids. Studies have shown that it is an effective non-invasive treatment with minimal associated risks as compared to myomectomy and hysterectomy. MRgFUS can be offered to a majority of patients suffering from symptomatic uterine fibroids. It has been suggested that the use of broader inclusion criteria as well as the mitigation techniques makes it possible to offer MRgFUS to a much larger subset of patients than previously believed. This paper will describe how MRgFUS treatment for uterine fibroids is performed at the University of Malaya Medical Centre, Kuala Lumpur, Malaysia.

## INTRODUCTION

Fibroids are benign growths in the uterus, which are symptomatic in up to 25 percent of women of childbearing age [1]. Symptoms can include heavy and prolonged menstrual bleeding, severe pain, bloating and constipation or urinary complaints. The most common treatment is hysterectomy, a highly invasive surgical procedure to remove the uterus, which is associated with the usual surgical risks and complications, requires a three- to four-day hospital stay and results in patient recovery time of six weeks or more [2,3]. All other techniques including uterine artery embolisation [4], involve some level of incision, hospitalization and recovery time. For example, myomectomy requires a hospital stay of several days and recovery time of two to four weeks [5]. A number of noninvasive alternatives to hysterectomy have become available as treatments for uterine fibroids [5-9].

MRgFUS is so disruptive that it will very likely overturn the other dominant current “technologies” e.g. surgery [10]. Clinical studies demonstrate that MRgFUS is a safe and effective treatment for symptomatic uterine fibroids [11-13]. Several studies have shown that MRgFUS significantly improves clinical symptoms in 70% to 80% of women with uterine myomas [13-16]. Studies have also demonstrated a correlation between the treated volume of the myomas, improvement in symptoms, and lesion shrinkage [12]. The results demonstrate that successful and durable treatment of uterine fibroids with MRgFUS necessitates selecting those patients for whom higher non-perfused volumes can be attained using the MRgFUS system.

In contrast to other invasive treatments for uterine fibroids, the relatively non-invasive MRgFUS can be performed as an outpatient procedure and requires no general anaesthesia. The ExAblate 2000 (InSightec Ltd., Haifa, Israel) is the first device to combine magnetic resonance imaging (MRI) with high-intensity focused ultrasound to destroy tumours non-invasively. In contrast to other options, the non-invasiveness of the MRgFUS technique is associated with minimal risks and complications, requires no overnight hospital stay and allows most patients to return to work and their normal activities in one to two days.

Safety data from these studies consistently show that there are few serious FDA-reportable adverse events related to MRgFUS. The only device-related adverse events reported especially early on were skin burns (secondary to poor coupling from hair and scars on the skin) and nerve damage following MRgFUS, which resolved within a year [15]. Damage to adjacent organs, such as bowel perforation, is also possible during treatment but rare [17]. To date, no immediate emergency surgical interventions, unexpected short-term adverse events or long-term complications have been observed after MRgFUS.

Based on the prospective registry of all known pregnancies occurring after MrgFUS, and maintained by the device manufacturer and reported to the FDA, 54 pregnancies in 51 women have occurred. The mean time to conception was 8 months after treatment. Live births occurred in 41% of pregnancies. There was a 28% spontaneous abortion rate, an 11% rate elective pregnancy termination rate and 20% ongoing pregnancies beyond 20 gestational weeks. The mean birth weight was 3.3 kg, and the vaginal delivery rate was 64%. [18]

It also has been suggested that for the NHS in the UK, a treatment strategy for symptomatic uterine fibroids starting with MRgFUS is likely to be cost-effective [21]. It also remains cost-effective under alternative assumptions regarding current practice, health utility estimates before and after treatment, and the effectiveness of alternative treatments (complication rates, recurrence rates and procedural death rates).

Currently, MRgFUS has been used for breast tumours, painful bony metastases, and liver tumours. For the brain, it has been used for the ablation of glioblastomas and for functional neurosurgery. Future applications for prostate cancer and acute stroke treatment are being explored.

All MRgFUS procedures were performed using the ExAblate 2000 (InSightec, Haifa, Israel), which is fully integrated with a 1.5 Tesla MR scanner (GE Medical Systems, Milwaukee, WI). ExAblate uses a ‘sonication’ process wherein focused ultrasound (FUS) destroys tissues by concentrating a high-energy beam on a specific point and raising its temperature to 60°-85°C. Multiple sonications (focal delivery of energy) are required to ablate a specific tissue. The MRI system provides critical data such as high-resolution 3D imaging of the location of the tumour and internal organs as well as real-time temperature feedback that indicates the degree of tissue heating and coagulation. Thus, this integration of FUS with MRI provides a “closed-loop therapy and feedback system” that enables the physician to adjust treatment parameters and control the treatment, helping to ensure a high level of safety and efficacy. This is currently not commercially possible on the ultrasound- based focused ultrasound systems.

## SCREENING MRI

If the patient is clinically eligible and interested in MRgFUS, she is referred for a screening MRI scan. Screening is performed in the prone position to flatten the abdomen (on a 1 cm gel pad similar to that used for treatment) and to allow the pelvic structures to fall into the position they would be in during a potential treatment. Imaging consists of several sequences. To identify anatomical structures within the pelvis, T2-weighted fast spin echo images in axial, sagittal and coronal orientations are acquired. To evaluate the presence of hemorrhagic or fatty tissues, T1-weighted fast spoiled gradient echo (FSPGR) images are acquired in the sagittal orientation followed by T1-weighted fat suppressed FSPGR images post gadolinium injection in order to evaluate the hemodynamic characteristics of the fibroids and to assess their potential viability.

## PATIENT SELECTION

The first, and probably, the most important inclusion criterion for selecting MRgFUS as a treatment is the existence of uterine fibroid(s) and the relevance of the fibroid(s) to the patient’s symptoms. The location and size of the fibroids must correlate with the patient’s symptoms. However, if a patient’s symptoms do not correlate with the size and location of the leiomyomas, MRgFUS may not be the appropriate treatment, for example, subserosal leiomyomas associated with uterine bleeding instead of compression symptoms to adjacent organs i.e. bladder and intestines [20]

The exclusion criteria are as listed in Table 1 [21]

**Table 1:** Patient exclusion criteria.

1. Hemoglobin <10 mg/dL
2. Patient has hemolytic anemia
3. Patient has unstable cardiac status including:
• Unstable angina pectoris on medication
• Documented myocardial infarction within six months of protocol entry
• Congestive heart failure requiring medication (other than diuretic)
• Currently taking anti-arrhythmic drugs
• Severe hypertension (diastolic BP > 100 on medication)
• Presence of cardiac pacemaker
4. Patient has severe cerebrovascular disease (multiple CVA or CVA within six months)
5. Patient is on anti-coagulation therapy or has an underlying bleeding disorder
6. Evidence of uterine pathology other than leiomyoma
7. Patient has an active pelvic infection
8. Patient has an undiagnosed pelvic mass outside the uterus.
9. Patient's weight >110 kg
10. Patient with extensive longitudinal abdominal scarring in an area of the abdomen directly anterior to the treatment area.
11. Patient with standard contra-indications for MR imaging such as non-MRI compatible implanted metallic devices.
12. Individuals who are not able or willing to tolerate the required prolonged stationary prone position during treatment (approximately 3 h.)

**Figure 1:**
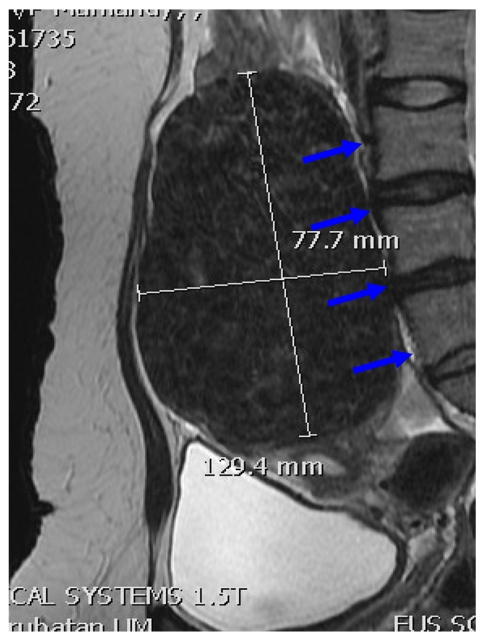
Sagittal T2-WI shows a “black” fibroid which is very close to the LS spine (blue arrows) and cannot be treated safely unless the bladder is drained or other mitigation techniques performed.

**Figure 2:**
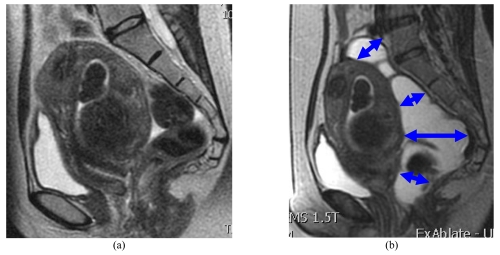
Sagittal T2-WI of the pelvis. Use of rectal filling (arrows) to displace the uterus away from the sacrum.

As bone absorbs ultrasound waves more readily than soft tissue, low energies are sufficient to heat a bone surface to high temperatures. Consequently, nerves lying adjacent to a heated bone surface may be heated resulting in pain and, in extreme cases, even result in nerve damage [15]. It is now recommended that sonications are performed at least 4 cm from bony structures to minimize the amount of heating of the bone [14] (Figure 1), which can in turn heat the fat surrounding the nerves and lead to stimulation or potentially damage of the nerve. Such stimulation of the adjacent sacral nerves may result in incomplete treatment with reduced efficacy of the procedure if the pain is severe. Therefore, fibroids lying close to the lumbosacral plexus or to any bone surface require special care in MRgFUS treatment. Several mitigation techniques such as tilting the beam path to avoid bone, increasing the frequency of the ultrasound beam, rectal filling to push the fibroid away from the bone [15] (Figure 2) or partial treatment to change the subsequent orientation/location of fibroid are available.

**Figure 3:**
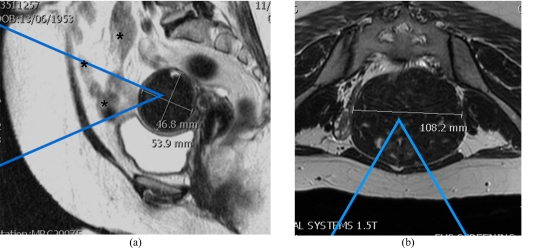
(a) Sagittal T2-WI shows no acoustic window (blue triangle represents the FUS beam path) secondary to bowel (asterisks) lying anterior to fibroid (may be overcome by filling the bladder and/or rectum) while (b) axial T2-WI shows a good acoustic window.

Patients are deemed technically suitable for MRgFUS if their fibroids mass seems accessible by the system (Figure 3) and is not deemed too large in volume. A significant proportion of the fibroids mass should be no more than 12 cm depth away from the skin line (which is the maximum depth of penetration of the sound). Fibroids with more than 50% of their volume beyond the maximum focus are generally excluded unless mitigation techniques are used, for example using a thinner acoustic coupling gel pad (reducing the distance between the patient and the transducer) or filling the rectum with ultrasound gel to push the uterus and the fibroids towards the anterior.

Patients with more than six uterine fibroids of more than 4 cm in size each should also possibly be excluded. This is generally associated with the fibroids being close to the sacrum or hidden behind the bowel and, thus, will be inaccessible. Pre-treatment of large fibroids with a gonadotropin-releasing hormone (GnRH) agonist helps to reduce fibroid volume and increase fibroid tissue susceptibility to the treatment, which may improve MRgFUS outcomes [22].

Presence of longitudinal scars in the beam path, including those that could not be seen on the MR images are also reasons for exclusion. Scar tissue may absorb the ultrasound energy and cause pain or result in a skin burn. The St Mary’s group has developed an ingenious method of highlighting transverse scars [21] where the scar is painted with a solution of nail varnish and paramagnetic iron oxide particles. This provides an obvious artefact along the line of the scar, which can easily be avoided by appropriate positioning and angling of the ultrasound beam.

**Figure 4:**
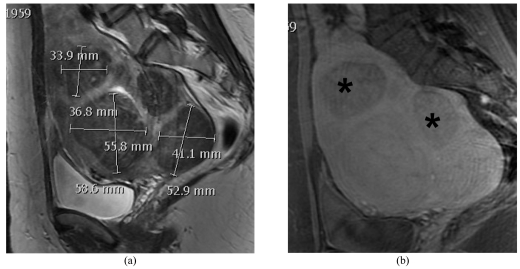
(a) Sagittal T2-WI shows multiple dark fibroids which on the (b) post contrast T1-WI shows some of the fibroids to be non-enhancing (*) and therefore need not be treated.

Other exclusion criteria include grossly calcified fibroids i.e. the pseudo capsule of the fibroid becomes heavily calcified where the ultrasound energy is disrupted by the capsular calcifications and cannot pass into the body of the fibroid mass. Non-enhancing fibroids, which are essentially non-viable, are also excluded. They may, however, be treated if they cause symptoms such as mass effect (Figure 4). Pedunculated fibroids, when attached by a small stalk, are another contraindication to MRgFUS as they may detach into the abdominal cavity, thus requiring further surgical interventions. Patients with other pelvic pathologies (such as adenomyosis) should not be treated with MRgFUS.

As air bubbles or hard particles may be present in the bowel and may reflect or absorb the ultrasonic energy, patients with bowel that cannot be potentially shifted from the beam path (by bladder or rectal filling) or beam angulation are also excluded from treatment.

What about adenomyosis? Symptoms of adenomyosis are very similar to those of fibroid [23] and both can occur in the same patient. A junctional zone width of more than 12 mm was defined as adenomyosis [24]. It is not uncommon to have patients referred for screening as fibroids but subsequently found to have adenomyosis (35% to 55%) after MR imaging [25].

**Figure 5:**
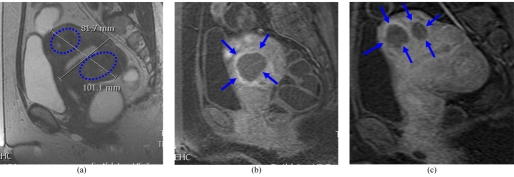
(a) Type A multiple area of focal adenomyosis (round) (blue dotted ring) seen in both anterior and posterior walls on the sagittal T2-WI; (b) and (c) post-contrast T1-WI following MRgFUS showing good response.

**Figure 6:**
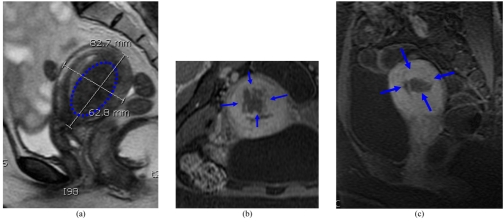
(a) Type B (serrated) area of focal adenomyosis seen in the posterior wall on the sagittal T2-WI; (b) axial and (c) sagittal post-contrast T1-WI following MRgFUS showing poor treatment (arrows) response with serrated margins.

The MRgFUS procedure can ablate adenomyosis tissue sufficiently and can improve symptoms significantly during a period of 3 to 6 months post-treatment [26] especially those with low-signal intensity adenomyosis on T2-weighted MR images. They went on to classify the architecture of the non-perfused lesions on contrast-enhanced T1-weighted MR images immediately after MRgFUS into 3 types: lesions with round margins (type R) (Figure 5), serrated margins (type S) (Figure 6), and honeycomb structures with numerous small, non-perfused holes (type H). There is still no long-term evidence currently available in print to demonstrate that treatment of adenomyosis results in clinical benefit.

## PATIENT PREPARATION

The patient’s abdomen is shaved and cleaned to remove any hair and also checked for the presence of any moles or scars as these may result in skin burns. A urinary catheter is inserted to control uterine movement during the three-hour treatment. An IV line is necessary for the administration of sedation. The patient lays prone over a water bath in which the transducer is immersed. The patient’s abdomen is acoustically coupled with the transducer via the water bath using a special gel pad. The patient's legs may be wrapped with compression stockings to reduce the risk of deep vein thrombosis but the authors do not practice this. Prior to starting the treatment, 100 mg Diclofenac sodium is given as a suppository. The patient is then positioned prone on the ExAblate treatment table with her abdomen over the water bath containing the ultrasound transducer. Patient’s blood pressure, heart rate, oxygenation, and comfort level are monitored throughout the treatment. The position of the patient over the transducer is determined from a 3plane localizer and T2-weighted imaging. This is to maximise the window available for the treatment of fibroids (images of poor positioning and images of baseline and MRI showing good positioning). Midazolam 2 mg is given via IV once the patient has been properly positioned on the FUS table. Pethidine 50 mg and Maxolon 20 mg are administered just prior to performing the sonications.

It is essential that the patient is comfortable in the position for the procedure. The authors ensure there is support for the head and arms, and provide headphones and ear plugs. Communication is provided through the 2 way-intercom and an emergency button. Analgesia is topped up regularly and the bladder, if filled to provide an acoustic window, is drained by 30-40 ml every 30 minutes to minimise the displacement of the uterus due to continued filling via the kidneys. The authors use updated MRI images to visualise the location of the fibroid in relation to the intended target fibroids (i.e. the center of the transducer) and make appropriate adjustments to the patient's position. The MRI scans also provide information as to the need to perform any mitigation techniques by changing the volume of the bladder, or filling the rectum with ultrasound gel using Sengstaken-Blakemore tube.

## TREATMENT PLANNING

**Figure 7:**
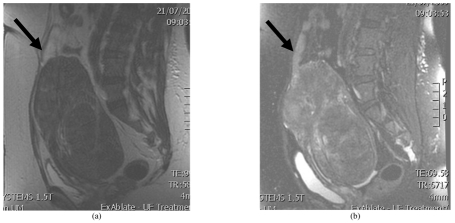
Defining the bowel on treatment planning (a) T2 WI in sagittal plane followed by T2WI with fat saturation shows the bowel (arrow) clearly against the suppressed intra-peritoneal fat.

When the position of the patient has been finalised and the patient is comfortable, there follows the acquisition of high-resolution T2-weighted MR images of the pelvis in the three orthogonal planes i.e. sagittal, axial and coronal. These images are used to define the location of the fibroid, the volume to be ablated, the proximity of the sacrum and lumbo-sacral spine as well as to determine the presence of any bowel lying anterior to the fibroid which may lie in the beam path. The authors have found that using a fat-suppressed T2-weighted imaging sequence assists in defining the intestine much easier (Figure 7).

If the fibroid lies too close to the sacral area, then ultrasound gel (approx 250cc) can be inserted rectally to displace the fibroids away form the area. A safe distance of 4 cm is necessary. If bowel has migrated anteriorly to the fibroid, then saline can be infused into the bladder. This may need to be followed with insertion of rectal gel. The skin-gel pad interface needs to be defined. This is followed by marking out the critical structures i.e. the pubic bone, bowel and far field bone (the spine and sacrum) using specific low-energy density region markers.

The region of treatment (ROT) is then defined on the targeted fibroid. This ROT is placed on the coronal images. The ROT is drawn such that a margin of at least 1 cm is kept from the serosal surfaces to minimise the risk of ablating the serosa which causes severe pain. There is no limit to the percentage of fibroid volume that can be treated though generally a 60-70% of ablation is necessary for good outcomes [13]. The operator then chooses the treatment plan depending on the size and type of fibroid being treated. For “white fibroids”, a nominal high-density plan should be selected, otherwise a medium-density plan would be suitable. The length of the sonications can then be selected and may vary from 10 mm to 45 mm. Based on the treatment plan chosen, the FUS system automatically displays a series of sonications to cover the region of treatment. Each spot is cylindrically shaped, 25 to 45 mm in length and 5 mm in diameter for a nominal fibroid.

The sonication beam path is carefully checked to ensure that it does not pass through any structures that should be avoided – such as the small bowel that can fall in front of the uterus.

Generally, treating and ablating an 8 cm fibroid takes approximately 3 h of sonications (that is excluding the patient preparation and planning), depending on energy absorption and location of the fibroid. Fibroids that are larger than 10 cm are less suitable for the treatment because of the long treatment time. However two options are available:

1. Treating the fibroid over two sessions performed preferably with the fibroid volume being split into a superior and an inferior region for each of the treatment. This is to avoid the ultrasound beam in the second treatment from passing through already treated regions if an anterior-posterior division was made.
2. Or as mentioned previously, use pre-treatment with GnRH agonist prior to MRgFUS to shrink the fibroid and improve treatment outcomes [15].

## TREATMENT

**Figure 8:**
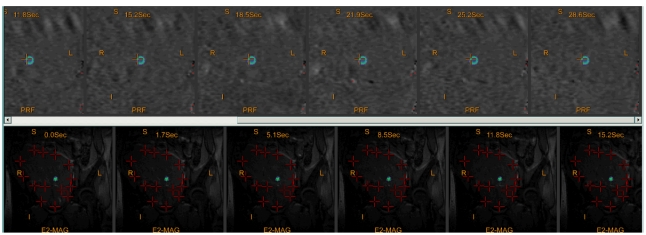
The verification sonication (blue ring) on the coronal image. The red crosses “X” are the fudicial markers to identify the uterus, pelvic bones, and bladder to ascertain if the patient has moved during the treatment.

Before treatment, the patient is asked to press the emergency button. One or more low-energy 'verification' sonications are then delivered to calibrate the location of the actual target spot against the planned target location (Figure 8). This is monitored on the temperature maps, which are displayed real time during the course of the sonications. These sonications also allow a titration of the energy to the patient progressively rather than being treated with a large dose in a single step.

The system will display the total number of sonication spots on the defined ROT. These spots are green, yellow or red (just like traffic lights) to indicate the safety of each sonication spot. The green spots can be safely treated while the yellow spots generally indicate that the energy density reaching the marked out areas of the sacrum has exceeded a system-defined lower safety limit. One can still treat these yellow spots but should try to modify the sonication parameters appropriately and ensure post sonication that the patients do not have discomfort in their back or radiation down their lower limbs. However, when the spots turn red, the system will not allow you to sonicate. This is generally due to areas of bowel, which lie within the beam pass zone and pass through the pubic bone or the energy density in the posterior pelvis reaching a system-defined upper margin. The beam must be angled to avoid the bowel, parameters changed or the spot moved.

**Figure 9:**
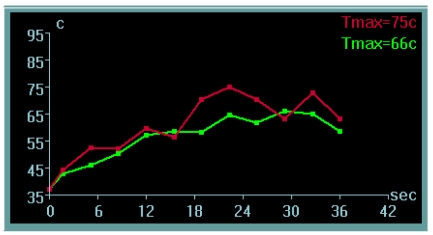
The post sonication temperature display of the tissue temperature in the fibroid after the treatment has been delivered.

**Figure 10:**
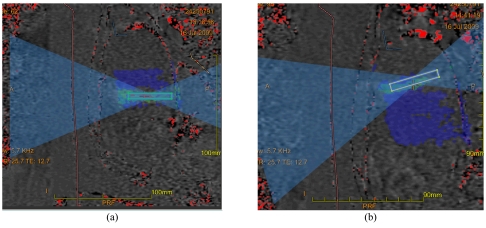
(a) The areas in dark blue are previous treatment areas that have achieved the desired thermal dose. The rectangular box (lighter blue) shows the current sonications application while the green area shows the current sonications dose map. (b) The inverted light blue cones show the beam path which can be angulated to avoid critical structures. The rectangular box (yellow) shows the current sonication has exceeded the recommended dose but can still be delivered. The areas of red are phase shift artefacts.

Each sonication duration varies from 20 to 30 seconds. This is followed by cooling periods of 24 to 90 seconds. The cooling periods allow time for the skin to cool down; otherwise patients may suffer skin burns. Thermal feedback is generated by real-time PRF while magnitude images highlight the temperature changes and the anatomy in the targeted area. A temperature graph shows the temperature change on the temperature maps (Figure 9). Once the targeted tissue reaches the desired temperature leading to thermal necrosis [27] system automatically highlights the treated areas in blue (Figure 10). The sonications can be monitored in axial, coronal or the sagittal plane.

For the patient’s safety, a cooling period between each sonication is programmed into the treatment plan, to allow cooling of tissue outside and anterior to the treatment zone. This cooling duration varies depending on the output energy and how close the next sonication is to the area just treated, but is generally between 40 and 70 seconds. Using an interleaved mode in large fibroids shortens the cooling time and hastens the treatment time where subsequent sonications anterior pass zones do not intersect.

**Figure 11:**
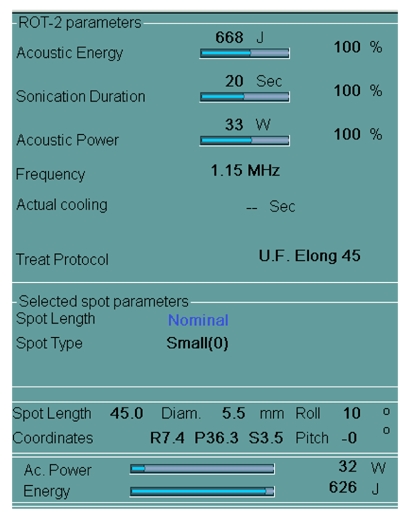
The system allows changes to the energy, frequency, spot parameters depending on the patient comfort and the response of the fibroids to the sonications. The system also displays the spot length, diameter of the sonication spot along with the angulation of the beam (roll and pitch).

Sonication parameters can be changed, if necessary, including energy, duration, spot size and frequency depending on the response seen in the previous treatment or due to pain or discomfort (Figure 11).

During each sonication, magnitude images, which display the position of the uterus relative to the electronic 3D special markers (fudicials) placed at the beginning of treatment on the T2 images, are obtained, and allows for determination of any movement of the uterus and patient.

**Figure 12:**
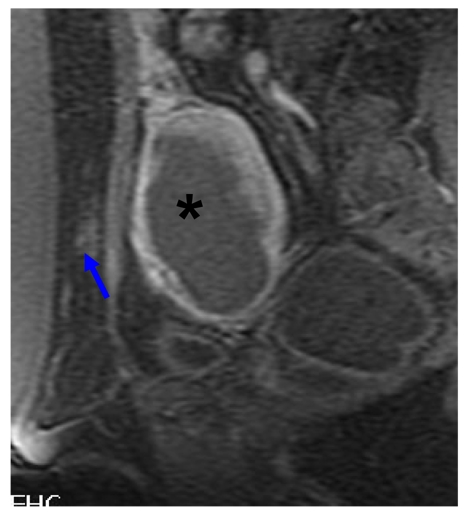
Post-contrast T1WI in sagittal plane showing enhancement of the subcutaneous tissue (arrow) and ablated region of fibroid (asterisk).

After all the sonications have been completed and the dose volume is determined to be adequate for fibroid destruction, the treatment has ended. Post-treatment fat-saturated T2WI are obtained to assess the skin and abdominal muscles for the presence of any hyperemia (Figure 12).

**Figure 13:**
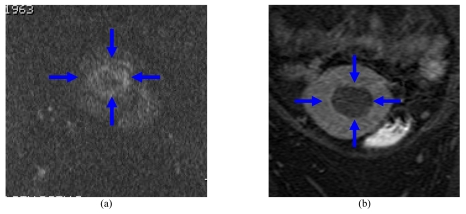
(a) Coronal DWI images compared to (b) post-contrast fat suppressed T1WI to assess the extent of ablation (arrows) at the same level.

DWI changes, which include changes in both T2 and ADC, may be useful in many cases to delineate the treated region resulting from MRgFUS. However, definite DWI changes are not always observed, and in some large treatments, the area of the non-perfused region may be under estimated [28]. Despite these limitations, these same sequences may be performed anytime during the treatment to assess damage or extent of ablation. The authors currently use axial DWI images using B values of 600 to assess the size of ablation (Figure 13).

**Figure 14:**
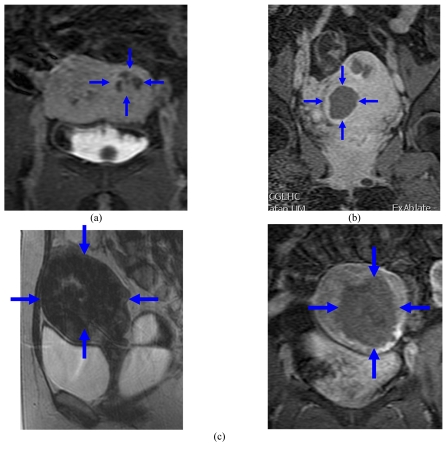
T1W post contrast enhanced images showing (a) poor (b) average and (c) good ablation (arrows).

A series of contrast-enhanced T1-weighted images are acquired to determine the treatment outcome, which shows the treated fibroid as non-enhancing (Figure 14). In the authors' practice, gadolinium contrast is not given intra-procedurally because there is a possibility that the gadolinium chelate will dissociate and the free gadolinium may become fixed in the tissues. The long-term effects of such exposure are not known. Therefore in the authors' centre, once the intravenous gadolinium has been given, the treatment has to be terminated.

Immediately following treatment, the patient’s skin is checked for the presence of any burns. In one study, approximately 11% of patients developed some inflammatory changes in the near field but these changes cause minimal if any post-treatment discomfort to the patients [29]. It is vital that the near field structures are monitored for heating on thermal images acquired during sonications. Occasional in-treatment acquisition of T2-weighted fat saturation images are helpful in detecting these changes early, especially if there is an awareness of the location of patient discomfort associated with sonications.

The authors do prescribe some post-procedure analgesics to take should patients experience any pain. Patients are provided with the contact phone number of the gynecologist should they need any assistance. Following treatment, the patient generally remains in the MRI suite for one hour to recover from the sedation. Then they are discharged and accompanied home by their spouse or a relative.

## MONITORING OUTCOMES

**Figure 15:**
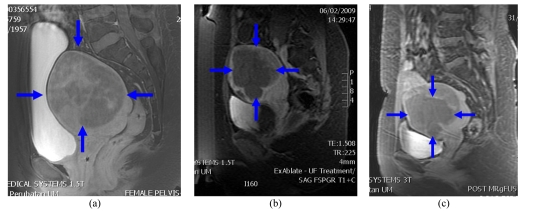
Fat saturated post-contrast T1WI (a) at baseline prior to treatment (b) following completion of ablation with area of non perfusion seen centrally and (c) at 6 months follow-up.

On the day following treatment, they should be able to return to their normal activities with no unusual events and no medication. The authors subsequently plan for them to visit the interventional radiology clinic in 2 weeks and see the gynecologist at the same time. Patients for whom treatment could not be completed because of the large size or number, a repeat treatment is planned anytime from 1 week onwards. Follow-up contrast-enhanced MRI is performed after 6 months to assess the fibroid size (Figure 15).

Patient-reported outcomes of symptom severity and Health-Related Quality of Life (HRQL) are becoming increasingly important for evaluating treatment of uterine fibroids within the clinical setting. The Uterine Fibroid Symptom and Health Related Quality of Life Questionnaire (UFS-QOL) is a uterine fibroid-specific questionnaire developed specifically to evaluate the symptoms of uterine fibroids and their impact on HRQL. The UFS-QOL appears to be a useful evaluative tool for assessing symptoms and HRQL in studies among patients with uterine fibroids [30]. The authors screen their patients using these criteria to assess response.

## SUMMARY

MR-guided focused Ultrasound provides an important new non-invasive and effective treatment for uterine fibroids. MRgFUS can be offered to the majority of patients suffering from symptomatic uterine fibroids [21]. They suggest that the use of broader inclusion criteria as well as the mitigation techniques makes it possible to offer MRgFUS to a much larger subset of patients than previously believed (Table 2). Recovery from the treatment is almost immediate and symptom relief is generally noticed sooner than alternative therapies. This is a tremendous advantage over existing options, which place a large burden on patients. Additional applications e.g. drug deliveries are under investigation and hold the promise of transforming the surgical arena to benefit millions of patients.

**Table 2:** Comparison of assessment criteria used in earlier studies and the current St. Mary’s centre [19].

**Clinical factors**	**Prior study**	**St. Mary’s study**
Insufficient symptoms of fibroids	SSS < 21 Excluded	Not relevant
Age < 40 or >60 years	Excluded	Not relevant
Desires pregnancy	Excluded	Not relevant
Menopausal	Excluded	Not relevant
Obesity >250Ibs	Excluded	Excluded
Prior UFE	Excluded	Not relevant
IUD, MR imaging incompatibility	Excluded	Excluded
**Technical factors**	**Prior study**	**St. Mary’s study**
Too much fibroid volume >900 cc	Excluded	Not relevant
Bowel obstructing beam path	Excluded	Partly mitigated
Significant adenomyosis	Excluded	Excluded
Pedunculated fibroids	Excluded	Excluded
Fibroids too small or no fibroids	Excluded	Excluded
Bright T2 fibroid	Excluded	Partly mitigated
Degenerating, necrotic, or infarcted fibroids	Excluded	Excluded
Arterial-venous malformation, calcified fibroids, or conglomerate of fibroids or septated fibroids hard to transmit heat across	Excluded	Excluded
